# Post-ejaculatory thermal sensitivity of sperm performance in the high-altitude lizard *Phymaturus extrilidus*

**DOI:** 10.1242/bio.062678

**Published:** 2026-06-30

**Authors:** Rodrigo Gómez Alés, Guadalupe López Juri, Franco Valdez Ovallez, Maximiliano Tourmente

**Affiliations:** ^1^ DIBIOVA (Gabinete Diversidad y Biología de Vertebrados del Árido), Departamento de Biología, Facultad de Ciencias Exactas, Físicas y Naturales, Universidad Nacional de San Juan, Av. Ignacio de la Roza 590 (O), Rivadavia, J5402DCS San Juan, Argentina; ^2^ CONICET (Consejo Nacional de Investigaciones Científicas y Técnicas), J5400ARL San Juan, Argentina; ^3^ Laboratorio de Biología del Comportamiento, Facultad de Ciencias Exactas, Físicas y Naturales, Universidad Nacional de Córdoba, X5000JJC Córdoba, Argentina; ^4^ Instituto de Diversidad y Ecología Animal, Consejo Nacional de Investigaciones Científicas y Técnicas (IDEA- CONICET, UNC), X5000JJC Córdoba, Argentina; ^5^ Centro de Biología Celular y Molecular, Facultad de Ciencias Exactas, Físicas y Naturales (FCEFyN-UNC), Universidad Nacional de Córdoba, X5016GCA Córdoba, Argentina; ^6^ Instituto de Investigaciones Biológicas y Tecnológicas, Consejo Nacional de Investigaciones Científicas y Técnicas (IIByT-CONICET, UNC), X5016GCA Córdoba, Argentina

**Keywords:** Global warming, Thermal sensitivity, Reproductive physiology, Sperm kinematics, Liolaemidae, *Phymaturus*

## Abstract

Rising environmental temperatures may differentially affect physiological processes in ectotherms, with sperm function representing a potentially critical but understudied vulnerability. We experimentally evaluated post-ejaculatory thermal sensitivity of sperm kinematics in the high-altitude viviparous lizard *Phymaturus extrilidus*. Ejaculated sperm from adult males were exposed to three ecologically relevant temperatures (28°C, 35°C, and 38°C), and motility and swimming parameters were quantified over a 2-h incubation period using computer-assisted sperm analysis. Sperm performance was strongly affected by temperature, incubation time, and their interaction, indicating progressive thermal damage. Contrary to our prediction, peak sperm motility and swimming performance occurred at 28°C rather than at the preferred body temperature (35°C). Incubation at 35°C and especially 38°C caused marked, declines in motility, curvilinear velocity (VCL), and average path velocity (VAP), while straight-line velocity (VSL) remained comparatively stable. Increased linearity and straightness at elevated temperatures suggest reduced trajectory complexity and flagellar oscillation amplitude. These results reveal a mismatch between the thermal optimum for sperm function and locomotor performance, highlighting a physiological trade-off between somatic and reproductive traits. Under climate-warming scenarios, reduced access to cooler microhabitats may accelerate sperm senescence and compromise reproductive success in high-mountain ectotherms.

## Teaser

**Summary:** Sperm performance in a high-altitude lizard peaks below preferred body temperature, revealing a thermal mismatch between reproductive and somatic performance and highlighting potential reproductive vulnerability under ongoing climate warming.

## INTRODUCTION

Global climate change is altering thermal conditions experienced by reptiles, potentially affecting ecological and physiological traits across species in this group (Sinervo et al., 2010; Huey et al., 2012; Kearney, 2013; Jara et al., 2019). Of particular concern, rising environmental temperatures may reduce the daily activity periods of organisms, thereby decreasing their opportunities for reproduction and foraging, which in turn can increase the risk of mortality or local extinctions (Sinervo et al., 2011, 2024; Lara-Resendiz et al., 2024; Wild et al., 2025). This is especially critical because body temperature governs key physiological and behavioral processes in ectotherms, including locomotion, reproduction, and growth (Sinervo and Dunlap, 1995; Angilletta et al., 2002; Gómez Alés et al., 2018; Quintero Pérez et al., 2023), while thermoregulation depends strongly on environmental conditions such as ambient temperature and humidity, rendering these organisms particularly vulnerable to climate change (Kingsolver et al., 2013; Paaijmans et al., 2013). To cope with these changes, reptiles employ a variety of thermoregulatory acclimation strategies that help buffer the effects of environmental temperature on physiological performance and minimize fluctuations in body temperature within a range of preferred temperatures (Adolph and Porter, 1993; Angilletta, 2009; Huey et al., 2009). Although several studies have focused on how reproductive traits of ectotherms respond to rising temperatures (Kingsolver et al., 2013; Wang and Gunderson, 2022), relatively few have addressed these responses in lizards (Licht and Basu, 1967; Dubey and Shine, 2011; Rossi et al., 2021; Domínguez-Godoy et al., 2024). Nevertheless, it has been suggested that viviparous lizard species may be more vulnerable to climate change than oviparous species (Sinervo et al., 2010; Wang et al., 2017; Jara et al., 2019).

Understanding reproductive traits in lizards is essential for interpreting their life history strategies and potential adaptations to environmental fluctuations, as well as for assessing species conservation status (Shine, 2004; Boretto et al., 2020). Within this broader reproductive context, male reproductive success is particularly environmentally sensitive. The male reproductive cycle is affected by temperature through multiple pathways: directly via thermal effects on physiological processes, and indirectly, through variables that may be influenced by temperature, such as synchronization with female reproductive cycles and the availability of receptive females within the population (Saint Girons, 1985; Méndez de la Cruz et al., 2014; Boretto and Ibargüengoytía, 2021; Valdez Ovallez et al., 2024). On one hand, rising environmental temperatures, combined with increased thermoregulatory precision, may accelerate testicular spermatogenesis in organisms inhabiting high-altitude environments with climatic constraints (Carretero et al., 2005; Castro et al., 2018; Gómez Alés et al., 2022). On the other hand, thermal stress may have deleterious effects on sperm physiology, ultimately reducing fertility (Méndez de la Cruz et al., 2014; Quintero Pérez et al., 2023). To date, studies evaluating temperature effects on sperm traits in reptiles remain limited, although existing work has examined both pre-ejaculatory and post-ejaculatory thermal influences (Wang and Gunderson, 2022; Quintero Pérez et al., 2023; Domínguez-Godoy et al., 2024). Pre-ejaculatory effects are primarily associated with damage to testicular tissue, where spermatogenesis occurs (Licht, 1965; Licht and Basu, 1967; Pearson et al., 1976), and to epididymal tissue, where sperm maturation takes place (Gist et al., 2000; Dosemane and Bhagya, 2015). Such damage can affect multiple traits including morphology, motility, DNA integrity, and the presence of cytoplasmic droplets (Quintero Pérez et al., 2023; Domínguez-Godoy et al., 2024). In contrast, post-ejaculatory thermal effects relate to the range of temperatures to which sperm are exposed in different environments before reaching the ovum, where their performance may also be influenced by interactions with the physicochemical conditions of the female reproductive tract (Tourmente et al., 2011; Gasparini et al., 2012; Rossi et al., 2021; Wang and Gunderson, 2022).

Among the various sperm characteristics that influence fertilization, morphological and dynamic traits are most commonly assessed in reptiles (Gage et al., 2004; Tourmente et al., 2006, 2007, 2008; López Juri et al., 2018; Blengini et al., 2020). Dynamic traits, including motility percentage and sperm velocity measures, are excellent indicators for understanding both competitive and non-competitive fertilization success across species (Froman et al., 1999; Gage et al., 2004; Tourmente et al., 2011). Faster sperm are expected to be more competitive during sperm competition, as they may reach the ovum more quickly than slower sperm (Snook, 2005; Tourmente et al., 2009). Importantly, previous studies have evidenced that dynamic traits are temperature-sensitive, with reduced motility or lower sperm velocity observed at temperatures of 37–38°C (Tourmente et al., 2011; Rossi et al., 2021). In addition, sperm from lizards exposed to preferred body temperatures for extended periods showed decreased performance (Quintero Pérez et al., 2023). Collectively, these findings suggest that studying sperm sensitivity to different environmental temperatures may be a valuable tool for assessing male fertility potential in reptile populations that are particularly vulnerable to environmental changes and anthropogenic habitat modifications.

The genus *Phymaturus* (Squamata: Liolaemidae) provides an ideal model system for such studies, since it includes 52 recognized species inhabiting cold and extreme environments in the high Andes of Argentina and Chile, as well as basalt plateaus in southern Argentina (Abdala et al., 2021). Remarkably, all species in the genus are primarily herbivorous, strictly saxicolous, viviparous, and exhibit annual or biennial reproductive cycles (Castro et al., 2018; Hibbard et al., 2019; Boretto et al., 2020; Boretto and Ibargüengoytía, 2021). Within this genus, *Phymaturus extrilidus*, belonging to the subclade *Phymaturus mallimaccii*, is a medium-sized and robust viviparous lizard with internal fertilization [maximum snout-vent length (SVL) of 103.9 mm] with an herbivorous diet (Abdala et al., 2021), which utilizes rocky outcrops, crevices, bare soil, shrubs, and wetlands during its daily activity (Gómez Alés et al., 2017). This species is endemic to the Sierra de la Invernada at 3100 m a.s.l. in the Puna region of Argentina (Lobo et al., 2012), where the climate is characterized by marked thermal heterogeneity, with low air temperatures and large daily temperature fluctuations (Sears et al., 2011; Gómez Alés et al., 2017, 2022; Vicenzi et al., 2021). The specific thermal conditions of these high-altitude and high-latitude environments, with wide variations and short activity seasons, impose particular challenges to the reproductive physiology of *Phymaturus* species (Boretto et al., 2010; Castro et al., 2018; Valdez Ovallez et al., 2024), potentially making sperm thermal sensitivity a crucial factor for reproductive success. Previous studies on the reproductive traits of *P. extrilidus* have demonstrated the existence of sexual dimorphism in size and shape, and suggest a biennial reproductive cycle in females synchronized with the annual and pre-nuptial cycle of males (Pizarro et al., 2022). Spermatogenesis in males occurs from spring to early autumn, and according to gonadal studies by Pizarro et al. (2022), mating takes place from late summer (March) to early autumn (April). However, a field observation of copulation in February reported by Noriega et al. (2023) suggests that mating activity may begin earlier in the season. From a thermoregulatory perspective, studies on the eco-physiological traits of *P. extrilidus* reveal that it has an average active body temperature of 32.3°C, which is lower than its preferred temperature (mean 35.7°C) determined under laboratory thermal gradients, with no intra-specific or seasonal variations observed in these traits (Gómez Alés et al., 2017). Despite inhabiting a harsh climate, *P. extrilidus* exhibits moderate thermoregulatory behaviour, successfully maintaining body temperatures close to its preferred temperatures, while exhibiting a wide thermal tolerance range (7–44°C; Gómez Alés et al., 2018).

In this context, our main objective was to experimentally evaluate post-ejaculatory thermal effect on sperm performance in male *Phymaturus extrilidus*. Specifically, we posed the following question: do sperm motility and velocity traits show sensitivity to different temperature treatments? To address this question, we exposed ejaculated sperm to three thermal treatments (28, 35, and 38°C), representing a range of optimal and suboptimal temperatures commonly experienced by males in their natural environment during the breeding season (Gómez Alés et al., 2017, 2018; Pizarro et al., 2022), which could increase under climate change scenarios. Our experimental design was based on evidence showing that while physiological performance is in many cases optimized at body temperatures close to the preferred temperature (T_pref_) of a species (Huey and Bennett, 1987; Angilletta et al., 2002; Dematteis et al., 2022), the preferred temperature range may not be equally favorable for all physiological processes, and could even be detrimental to some (Méndez de la Cruz et al., 2014; Gómez Alés et al., 2018; Quintero-Pérez et al., 2023). Based on these considerations, we hypothesized that post-ejaculatory thermal variation would affect sperm performance in *P. extrilidus* males, predicting that sperm motility and velocity traits would be optimal near the species' preferred body temperature (35°C) and would decline at higher temperatures (38°C), consistent with patterns reported in other squamate species (Tourmente et al., 2011; Rossi et al., 2021).

## RESULTS

Semen samples were obtained from all 22 individuals. However, four individuals produced samples with insufficient sperm concentrations to complete analyses across all temperature treatments and time points. Therefore, the final dataset comprised 18 males with complete kinematic data for all experimental conditions. Descriptive statistics (mean±standard deviation) for all sperm parameters are provided in Table 1.

**Table 1.** Summary of *P. extrilidus* sperm motility and velocity parameters at different temperatures and incubation times

Temperature	Time (min)	MOT (%)	VCL (μm s^−1^)	VSL (μm s^−1^)	VAP (μm s^−1^)	LIN (%)	STR (%)	WOB (%)	BCF (Hz)	Fractal
28°C	0	87.56±6.92	43.85±3.58	18.82±3.17	38.69±3.11	43.67±6.57	48.99±6.07	88.22±2.55	2.82±0.16	1.44±0.11
	30	86.14±7.04	46.01±4.45	21.51±3.23	41.01±4.12	47.52±5.80	52.72±5.14	89.00±2.78	2.77±0.16	1.39±0.09
	60	81.72±14.53	43.49±6.55	20.39±4.04	38.61±5.98	48.13±7.33	53.55±6.92	88.75±2.89	2.77±0.23	1.40±0.10
	90	85.68±6.54	42.28±5.18	19.57±2.63	37.65±4.19	47.78±7.65	52.88±7.38	89.18±2.04	2.80±0.19	1.41±0.11
	120	84.40±6.74	42.19±6.46	20.25±3.34	38.06±5.23	49.47±10.07	54.08±9.43	90.42±2.63	2.75±0.21	1.37±0.10
35°C	0	87.02±5.57	42.22±4.32	19.38±3.01	37.34±3.71	47.08±7.18	52.60±6.63	88.50±2.63	2.74±0.23	1.41±0.10
	30	85.85±5.14	41.82±4.07	19.19±3.47	37.13±3.38	47.31±8.84	52.56±8.15	88.88±2.89	2.75±0.18	1.40±0.11
	60	83.57±7.42	40.07±4.83	20.13±4.66	35.71±4.53	51.63±9.89	57.02±9.24	89.20±3.17	2.74±0.16	1.36±0.12
	90	81.04±9.04	37.43±5.02	18.92±3.85	33.66±4.37	52.85±10.84	57.98±10.56	90.03±2.40	2.68±0.19	1.34±0.12
	120	78.02±10.69	32.48±6.12	17.95±3.83	29.17±5.21	58.02±11.23	63.55±11.00	90.08±2.16	2.49±0.33	1.30±0.10
38°C	0	88.30±5.20	41.17±4.39	19.68±4.05	36.49±3.68	48.78±8.78	54.39±8.39	88.58±2.61	2.71±0.18	1.38±0.11
	30	83.25±8.25	38.50±3.85	19.77±5.02	34.33±3.23	52.95±12.91	58.43±12.45	89.19±3.08	2.73±0.18	1.35±0.14
	60	81.12±11.19	35.82±3.90	19.84±3.66	32.18±3.15	57.49±11.57	63.01±10.94	89.94±2.62	2.67±0.21	1.31±0.12
	90	78.88±17.85	32.44±5.50	18.13±4.63	29.20±4.85	58.32±12.69	63.87±11.97	90.06±2.87	2.57±0.23	1.29±0.12
	120	69.34±19.21	28.44±4.08	16.61±3.63	25.65±3.59	60.49±11.94	66.39±11.60	90.01±2.20	2.45±0.23	1.27±0.11

Values are presented as mean±standard deviation (*n*=18). MOT, percentage of motile cells; VCL, curvilinear velocity; VSL, straight-line velocity; VAP, average path velocity; LIN, linearity; STR, straightness; WOB, wobble coefficient; BCF, beat cross frequency; Fractal, fractal dimension.

Both temperature and incubation time had significant effects on all nine sperm parameters examined (Table 2). Importantly, significant temperature×time interactions were detected for seven of nine parameters with two exceptions: straight-line velocity (VSL) and the wobble coefficient (WOB), which showed no significant interactions (VSL: *X^2^*=15.42, *p*=0.052; WOB: *X^2^*=9.96, *p*=0.268).

**Table 2.** Statistical analyses of the effects of incubation time and temperature, and their interaction on sperm parameters in *P. extrilidus* (*n*=18)

Sperm parameters	Time		Temperature		Interaction	
	*X^2^*	*P*	*X^2^*	*P*	*X^2^*	*P*
MOT	453.46	**<0.001**	68.81	**<0.001**	161.98	**<0.001**
VCL	136.24	**<0.001**	156.26	**<0.001**	45.95	**<0.001**
VSL	18.30	**0.001**	10.80	**0.005**	15.42	0.052
VAP	108.06	**<0.001**	137.74	**<0.001**	42.89	**<0.001**
LIN	119.50	**<0.001**	110.22	**<0.001**	26.20	**<0.001**
STR	113.48	**<0.001**	116.42	**<0.001**	29.50	**<0.001**
WOB	67.79	**<0.001**	10.18	**<0.006**	9.96	0.268
BCF	73.67	**<0.001**	56.69	**<0.001**	32.16	**<0.001**
Fractal	98.41	**<0.001**	90.00	**<0.001**	21.27	**0.006**

MOT, percentage of motile cells (%); VCL, curvilinear velocity (μm s⁻¹); VSL, straight-line velocity (μm s⁻¹); VAP, average path velocity (μm s⁻¹); LIN, linearity (%); STR, straightness (%); WOB, wobble coefficient (%); BCF, beat-cross frequency (Hz); Fractal, fractal dimension. Chi-square (*X*^2^) values and associated *P*-values are shown for each fixed effect. Bold *P*-values denote statistical significance at α=0.05.

These interaction patterns indicate that the temporal trajectories of most sperm kinematic traits differed among temperature treatments.

### Sperm motility and velocity parameters

**Fig. 1.**
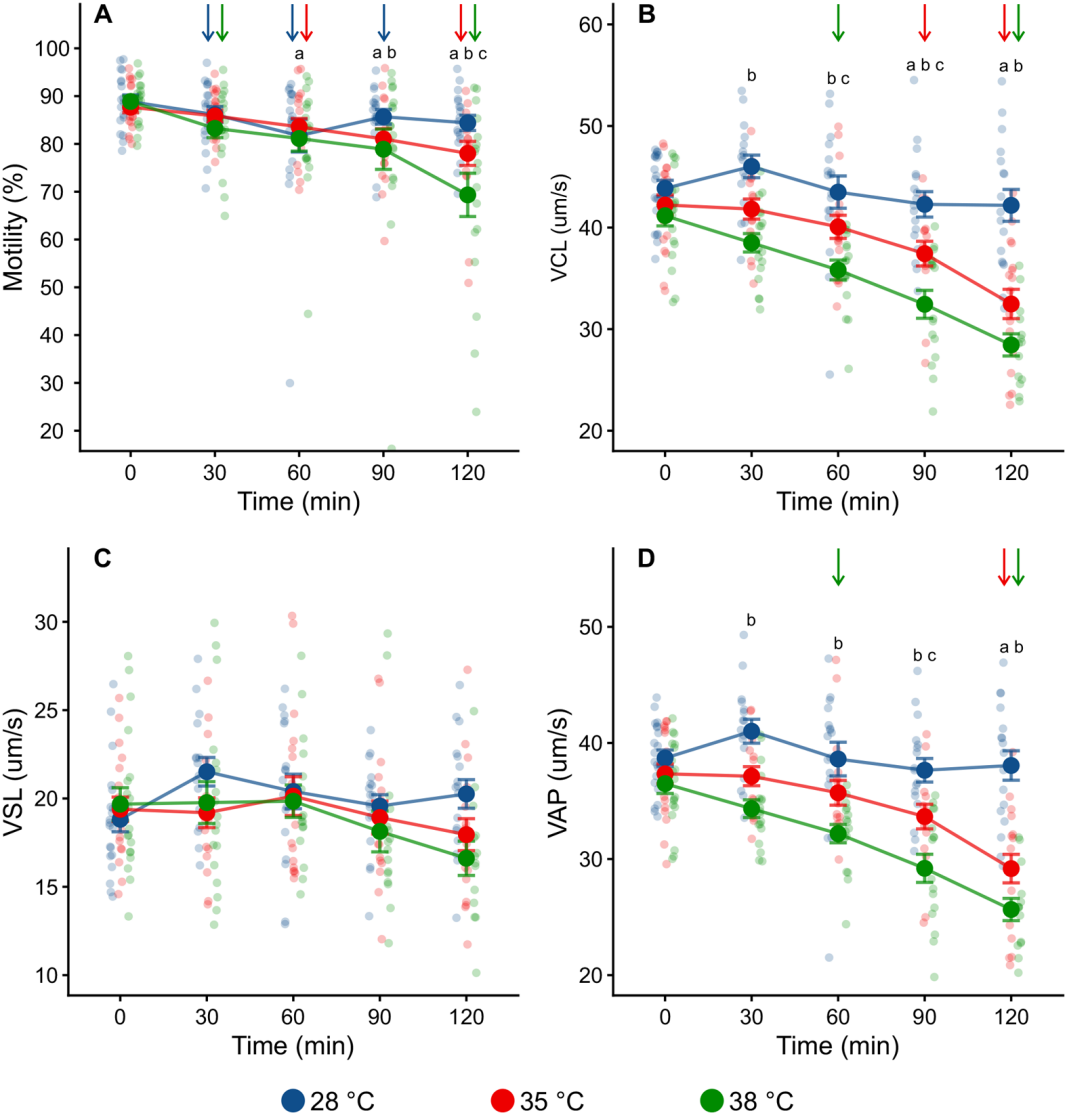
**Temporal dynamics of sperm motility and velocity parameters across three incubation temperatures in *P. extrilidus*.** (A) MOT, (B) VCL, (C) VSL, and (D) VAP. Incubation temperature: 28°C, blue; 35°C, red; 38°C, green. Solid points represent mean±s.e. (*n*=18 males). Transparent points represent individual data. Colored arrows indicate significant temporal changes within each temperature treatment (post-hoc test, *P*<0.05). Lowercase letters indicate significant differences among temperature treatments at each time point (post-hoc test, *P*<0.05): a, 28°C differs from 35°C; b, 28°C differs from 38°C; c, 35°C differs from 38°C.

Temperature and incubation time both significantly influenced the percentage of motile cells (MOT) and sperm swimming velocity parameters [VSL, curvilinear velocity (VCL) and average path velocity (VAP)] (Fig. 1). Sperm motility (%) showed significant temporal variation that was strongly modulated by temperature treatment (Fig. 1A). At 28°C, motility percentages remained high throughout the incubation period, except for a temporary dip at 60 min. At 35°C, motility decreased substantially (11% reduction) during the incubation period, with significant within-treatment temporal changes appearing at 60 min. The most pronounced motility decline occurred at 38°C, where the proportion of motile cells showed a 21% decrease from 0 to 120 min, with the first significant within-treatment drop appearing at 30 min. Between-treatment comparisons revealed that motility percentages diverged significantly beginning at 90 min, at which point the samples incubated at 35°C and 38°C exhibited significantly lower motility than those incubated at 28°C. By 120 min, all three treatments differed significantly from one another, with rank order 28°C>35°C>38°C.

VCL and VAP exhibited marked thermal sensitivity, with similar patterns for both parameters (Fig. 1B,D). At 28°C, VCL and VAP remained stable throughout the incubation period, whereas both parameters declined progressively over time at 35°C and 38°C. By 120 min, VCL and VAP at 35°C had decreased 21% from the initial value. The decline was even more pronounced at 38°C, where these parameters showed a 31% (VCL) and 29% (VAP) reduction from baseline. Post-hoc pairwise comparisons revealed that velocity differences among temperature treatments emerged as early as 30 min post-incubation, with the 28°C treatment differing significantly from 38°C. By 90 min, both elevated temperature treatments (35°C and 38°C) differed significantly from the 28°C treatment in both parameters, and this differentiation intensified through the final observation period.

In contrast to VCL and VAP, VSL showed considerably less thermal and temporal variation (Fig. 1C). VSL values ranged from approximately 19 to 20 μm s^−1^ across most treatments and time points, with only the 38°C treatment at 120 min showing a significant difference from the 28°C treatment. The temperature x time interaction was not statistically significant for this parameter (Table 2), and the magnitude of the changes was substantially smaller than those observed for the other velocity variables (Table 1), suggesting a comparatively weak thermal modulation over time relative to the other velocity variables.

### Sperm trajectory characteristics

**Fig. 2.**
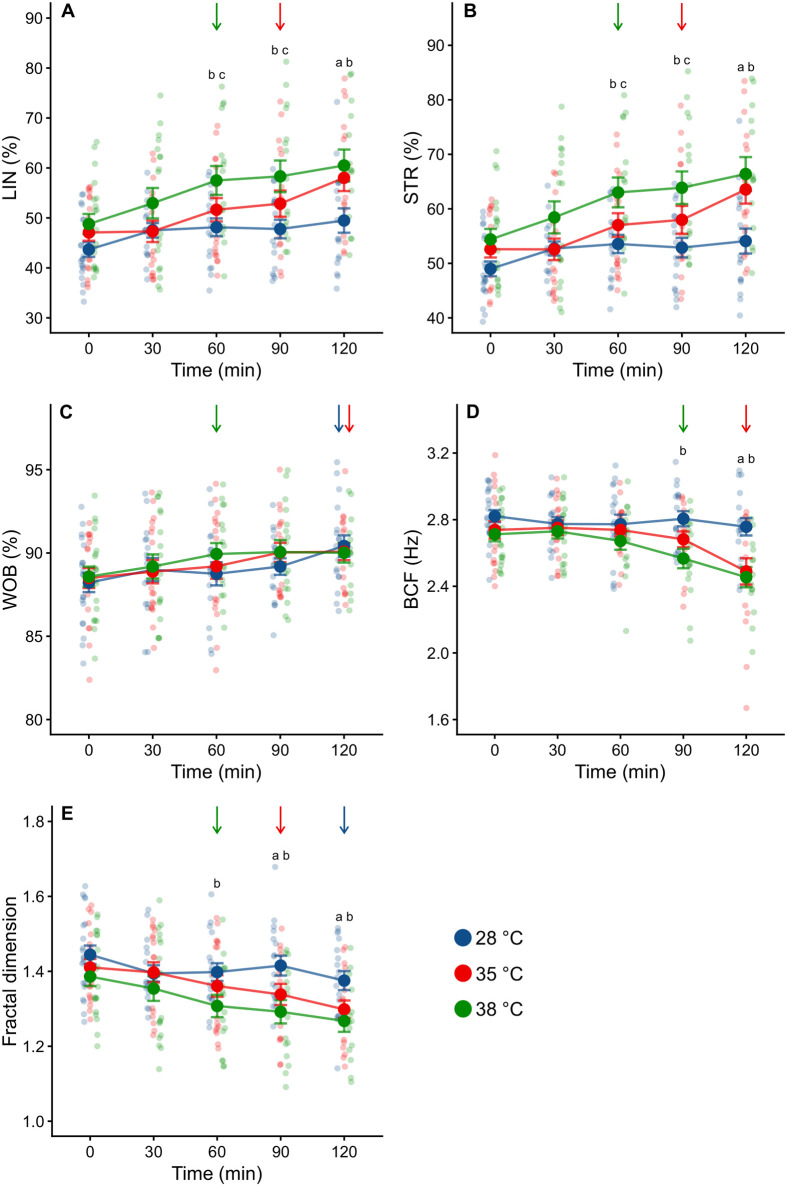
**Temporal dynamics of sperm trajectory shape parameters across three incubation temperatures in *P. extrilidus*.** (A) LIN, (B) STR, (C) WOB, (D) BCF, and (E) fractal dimension (FRACTAL). 28°C, blue; 35°C, red; 38°C, green. Solid points represent mean±s.e. (*n*=18 males). Transparent points represent individual data. Colored arrows indicate significant temporal changes within each temperature treatment (post-hoc test, *P*<0.05). Lowercase letters indicate significant differences among temperature treatments at each time point (post-hoc test, *P*<0.05): a, 28°C differs from 35°C; b, 28°C differs from 38°C; c, 35°C differs from 38°C.

Temperature and incubation time significantly affected all parameters describing the geometric properties of sperm swimming trajectories (Fig. 2). Linearity (LIN) and straightness (STR) both showed increasing trends over the 120 min period both at 35°C (LIN: 24%, STR: 19%) and 38°C (LIN: 22%, STR: 21%), while remaining relatively stable at 28°C (Fig. 2A,B). Significant between-treatment differences for both LIN and STR emerged at 60 min, primarily between the 28°C and 38°C treatments, with additional differentiation between 28°C and 35°C becoming apparent by 90 min. WOB increased slightly (∼2.5%) over time across all temperature treatments (Fig. 2C). Notably, the temperature×time interaction for WOB was non-significant, indicating that the temporal changes in this parameter did not differ among thermal treatments.

Beat-cross frequency (BCF) declined significantly over time, with this pattern being most pronounced at elevated temperatures (Fig. 2D). At 28°C, BCF remained relatively stable throughout the incubation period. In contrast, this parameter showed a 9% and 11% reduction at 35°C and 38°C, respectively. Significant between-treatment differences emerged by 90 min, when BCF at 28°C exceeded that at 38°C, with this differentiation persisting through the final time point.

Fractal dimension exhibited a decreasing trend over time that was markedly temperature-dependent (Fig. 2E). While, at 28°C, this parameter only decreased slightly at 120 min, at 35°C and 38°C the decline was more intense and sustained throughout the incubation period. The declining fractal dimension at elevated temperatures reflects simplified trajectory geometry, indicating that sperm swim in less complex, more constrained patterns at higher temperatures. Between-treatment differences for fractal dimension emerged at 60 min and became more extensive by 90 and 120 min, when both 35°C and 38°C treatments differed significantly from 28°C.

## DISCUSSION

Our results suggest that sperm performance in *P. extrilidus* is highly sensitive to post-ejaculatory thermal conditions, in agreement with our overarching hypothesis. Crucially, the significant temperature×incubation time interactions observed for most sperm parameters reveal that thermal damage accumulates progressively rather than acting as an instantaneous effect. However, the thermal optimum was not consistent with our initial prediction since sperm motility and velocity traits did not exhibit peak performance at the species' preferred body temperature (35°C; Gómez Alés et al., 2017). Instead, the highest and most stable performance across all sperm swimming and trajectory parameters was observed at 28°C, whereas incubation at both 35°C and particularly 38°C resulted in marked declines in sperm kinematic performance as incubation progressed. These findings indicate that thermal optima for sperm function do not necessarily coincide with temperatures that maximize other physiological processes, such as locomotor performance or general activity, highlighting a potential trade-off among different functional traits (Martin and Huey, 2008; Tourmente et al., 2011; Quintero Pérez et al., 2023; Caballero Vázquez et al., 2025). In this sense, Gómez Alés et al. (2018) provide evidence of thermal sensitivity in the locomotor performance of *P. extrilidus*, with optimal temperatures for maximum sprint speed (mean 32.5°C) being close to its active body temperature. Collectively, our results suggest that, after ejaculation, sperm function operates within a narrower and strongly time-dependent thermal window than general organismal performance.

The decline in sperm performance at elevated temperatures likely reflects direct physiological constraints on sperm cell integrity and energy balance, a pattern consistently documented across temperate and montane ectotherms (Wang and Gunderson, 2022; Domínguez-Godoy et al., 2024, 2025; Wang et al., 2025). In our study, the progressive deterioration of sperm motility and velocity at 35°C and 38°C is consistent with patterns observed in the lizard *Sceloporus aeneus*, where peak sperm performance occurs well below both the organism's preferred body temperature and locomotor optimum (Quintero Pérez et al., 2023). However, unlike that study, our results demonstrate that in the high-altitude viviparous lizard *P. extrilidus*, sperm deterioration accumulates progressively through time and affects not only sperm velocity but also trajectory geometry, revealing a strong temporal component of post-ejaculatory heat stress under ecologically relevant temperatures. Similarly, Rossi et al. (2021) reported in *Tropidurus spinulosus* a marked decline in sperm motility and in VCL and VSL as temperature increased from 34°C to 38°C.

Physiologically, this deterioration is associated with heat-induced acceleration of metabolic rates, which prematurely depletes the finite intracellular energy reserves of the gamete and promotes the accumulation of reactive oxygen species (ROS) (Hamilton et al., 2016; Capela et al., 2022; Gao et al., 2022). This oxidative imbalance can lead to lipid peroxidation, mitochondrial dysfunction, and ultimately reduced sperm motility and viability (Alavi and Cosson, 2005; Tourmente et al., 2011; Shahat et al., 2020; Wang and Gunderson, 2022). Furthermore, Domínguez-Godoy et al. (2024) showed that behavioral thermoregulation allows lizards to maintain body temperatures within the optimal range for sperm viability, potentially preventing the deleterious effects of sustained thermal stress on gamete function.

Our results provide evidence of temperature-induced stress in *P. extrilidus* sperm, manifested through specific alterations in swimming kinematics. The relative stability of VSL, contrasted with pronounced declines in VCL and VAP (Fig. 1), indicates that thermal stress primarily affects the lateral components of sperm swimming motion, most notably head yawing, rather than their progressive displacement component. This biomechanical distinction explains why sperm trajectories become progressively more linear and less oscillatory as thermal stress accumulates, as reflected by the increase in LIN and STR at 38°C (Fig. 2). When a similar pattern was reported in *T. spinulosus*, increased linearity under high-temperature conditions was interpreted, based on previous studies, as potentially facilitating more direct movement toward fertilization or storage sites (Rossi et al., 2021). However, in light of our results, this pattern more likely reflects a constraint imposed by thermal stress rather than an adaptive response.

This interpretation is further supported by comparisons with mammalian sperm physiology. In mammals, sperm trajectories become more linear as flagellar beating shifts from irregular to coordinated during epididymal maturation, with increased LIN and STR (Soler et al., 1994). In contrast, capacitation and hyperactivation, processes essential for fertilization, are associated with a marked reduction in linearity and the emergence of more vigorous, non-linear motility (Soler et al., 1994). Similarly, the decrease in BCF and fractal dimension suggests that thermal stress simplifies swimming geometry, reducing the spatial complexity of sperm trajectories (Katz and George, 1985; Mortimer et al., 1996) and potentially limiting the exploratory capacity of sperm cells. While moderate temperature changes can transiently increase trajectory complexity as part of thermotactic behavior (Boryshpolets et al., 2015), sustained thermal exposure appears to produce the opposite effect, consistent with a reduction in kinematic variability under prolonged thermal stress (Capela et al., 2022).

These patterns gain additional relevance when placed within the real thermal landscape experienced by reproducing individuals. In the harsh Puna environment, *P. extrilidus* faces a constant thermal contrast: while operative environmental temperatures average cooler values (∼24–32°C), thermoregulatory behavior drives individuals to actively seek their preferred temperature (∼35.7°C) to optimize other functions such as locomotion and digestion (Gómez Alés et al., 2017, 2018). Importantly, sperm maintained at 28°C (a temperature representative of operative environmental conditions experienced in the field) exhibited stable performance across all parameters throughout the observation period, indicating minimal functional stress under ecologically relevant thermal conditions. Sperm performance often declines at or slightly above the male's preferred or field-active temperature, revealing a trade-off between whole-organism performance (e.g. locomotion) and sperm quality (Quintero Pérez et al., 2023; Wang et al., 2025). As a consequence, sustained operation near voluntary thermal maxima would expose gametes to cumulative deterioration that could reduce their viability prior to fertilization (Rossi et al., 2021; Quintero Pérez et al., 2023), unless buffered by effective thermoregulatory behavior (Huey et al., 2009; Buckley et al., 2015; Gómez Alés et al., 2017; Domínguez-Godoy et al., 2024).

Our experimental design simulated short-term post-ejaculatory conditions in male *P. extrilidus*, by exposing sperm to a supportive extracellular medium under controlled temperatures. However, this approach does not incorporate direct interaction with female reproductive fluids, which could substantially modify the thermal responses observed. According to Gómez Alés et al. (2017), both males and females maintain body temperatures close to 32°C, a value already above the sperm thermal optimum detected here. Studies in *T. spinulosus* demonstrate that oviductal fluid can mitigate the negative effects of heat, improving sperm motility and velocity even under supra-optimal temperatures (Rossi et al., 2021), and whether an equivalent buffering mechanism operates in the viviparous reproductive tract of *P. extrilidus* remains an important open question.

Moreover, if *P. extrilidus* sperm exhibit similar thermal sensitivity during pre-ejaculatory storage, prolonged maintenance of supra-optimal body temperatures throughout the reproductive season could accelerate sperm senescence and reduce reproductive success. Given that reproduction in this species occurs toward the end of the activity season (Pizarro et al., 2022), sperm thermal sensitivity may represent a critical reproductive vulnerability under climate-warming scenarios (Wang et al., 2025). Future work should therefore explore pre-ejaculatory thermal effects on male reproductive physiology, as sustained exposure of the testes and epididymal environment to elevated body temperatures could further compromise sperm quality before ejaculation, consistent with early evidence that even mild thermal elevations can damage reptilian testicular tissue (Licht, 1965; Licht and Basu, 1967; Quintero Pérez et al., 2023).

In conclusion, our results have implications for understanding how reproductive traits, particularly sperm traits, may respond to climate warming in ectothermic vertebrates, especially in high-mountain specialist species. The genus *Phymaturus*, categorized as Vulnerable (Abdala et al., 2012), and *P. extrilidus* in particular, exhibits a slow life-history strategy characterized by delayed maturity, high longevity, and low annual reproductive investment (Boretto et al., 2020; Boretto and Ibargüengoytía, 2021; Pizarro et al., 2022), traits that likely amplify sensitivity to rapid environmental change. Our findings suggest the existence of a critical physiological trade-off: temperatures close to the field-active body temperature and preferred temperature (∼35°C), which optimize locomotor performance and behaviors associated with sexual selection, simultaneously act as cumulative stressors that progressively deteriorate sperm integrity and may ultimately compromise fertility. In this context, the availability of cooler microhabitats may be essential to buffer sperm thermal stress, allowing individuals to behaviorally regulate body temperature in ways that preserve gamete function. Under climate-warming scenarios (IPCC, 2018), the loss of thermal heterogeneity and the homogenization of environmental temperatures may reduce access to such microhabitats, increasing exposure to supra-optimal conditions, accelerating sperm senescence, and shortening the temporal window available for successful fertilization. Given that reproduction in *P. extrilidus* is already constrained by biennial female reproductive cycles and a male-biased operational sex ratio (Pizarro et al., 2022), thermally driven sperm deterioration represents a potential direct threat to population persistence. Taken together, our results suggest that the thermal safety margin of reproductive processes may be considerably narrower than that of general organismal functions, positioning male gametes as a critical vulnerability point for predicting ectotherm responses to climate change.

## MATERIALS AND METHODS

### Study site and fieldwork

We conducted fieldwork at the ‘Don Carmelo’ Private Reserve of Multiple Uses, located in the Sierra de La Invernada, west of the Ullum Department, in the Andes Mountain Range of San Juan Province, Argentina (30° 56.99′ S, 69° 04.83′ W; 3166 m a.s.l.). This area represents the southernmost edge of the desert Puna, situated between 2700 and 3400 m a.s.l. (Martínez Carretero, 1995). The climate is arid and cold, with an annual average temperature below 8°C and characterized by large daily and seasonal fluctuations in temperature (Gómez Alés et al., 2022). Precipitation is scarce (annual average 100 mm), occurring primarily in the summer, with typical snowfall from May to October (Martínez Carretero, 1995; Gómez Alés et al., 2022).

During February 2022, corresponding to the summer (reproductive) season, we captured 22 adult male individuals of *P. extrilidus*. Males were identified based on coloration traits, the presence of precloacal pores, and hemipenes eversion (Gómez Alés et al., 2018). Individuals were considered adults if their SVL equaled or exceeded the minimum reproductive size reported for the species (85 mm; Pizarro et al., 2022). Lizards were captured by hand or using a noose during their daily activity period, between 10:00 and 19:00 h (Gómez Alés et al., 2017). For each lizard, body temperature (T_b_) was measured using a thermocouple probe (TES TP-K01; 1.62 mm diameter) inserted 0.5 cm into the cloaca, within 10 s of capture to minimize heat transfer from the handler (Gómez Alés et al., 2017). For each individual we also recorded SVL (mm) using a digital caliper (SC111001, Schwyz RM, Argentina; ±0.02 mm) and body mass (g) using a digital scale (CH02, Diamond Premium MR, China; ±0.1 g).

### Laboratory experiments

Captured male lizards (*n*=22) were individually transported in cloth bags to the laboratory of Biología del Comportamiento, Universidad Nacional de Córdoba (Córdoba, Argentina). Lizards were housed individually in plastic enclosures (30 cm×40 cm×40 cm) with access to rock shelters, typically around 25–30 cm in size, and basking sites, maintained at 28°C on 10:14 h (light: dark) photoperiod to facilitate thermoregulation (Camacho and Rusch, 2017). All individuals received water *ad libitum*. Lizards maintained under these conditions for 8 days, during which sperm samples were collected. Following sample collection, all individuals were released in good condition at their original capture sites. All procedures were conducted in accordance with the guidelines for the management of amphibians and reptiles in field and laboratory research (Beaupre et al., 2004) and the regulations of Argentine National Law N° 14346. Collection and housing were authorized by the Secretaría de Medio Ambiente, Dirección de Conservación y Áreas Protegidas, Provincia de San Juan (exp. no. 1300-0031-22, R.G.A.).

### Sperm collection and sample preparation

Semen was collected from all males using electroejaculation according to López Juri et al. (2018). The electroejaculation protocol consisted of three series of five electrical stimuli, with each stimulus lasting 5 s. Rest periods were provided between successive stimuli and between series to allow muscular relaxation. This technique successfully induced semen expulsion into the cloacal ampulla in all individuals (100% success rate). However, although ejaculates were obtained from all 22 males, only 18 individuals produced samples with sufficient sperm concentration and volume to complete analyses across all temperature treatments and time points, and therefore all subsequent analyses were conducted using data from these 18 males.

Immediately following expulsion, semen was collected from the cloaca by adding 10 μl of sperm culture medium directly to the expelled sample and aspirating the semen-medium mixture with a micropipette. This mixture was then transferred to a microcentrifuge tube containing an additional 20 μl of medium. The resulting combination, hereafter referred to as the sperm suspension, was used to prepare samples for thermal treatment analysis. The composition of the sperm culture medium was: 108.30 mM NaCl, 25 mM NaHCO_3_, 4.80 mM KCl, 1.30 mM CaCl_2_.2H_2_O, 1.20 mM KH_2_PO_4_, 1.20 mM MgSO_4_.7H_2_O, 45 mM Hepes, 25 μg/ml gentamicin, 4% W/V bovine serum albumin (BSA), 10 mM glucose, and 2 mM sodium pyruvate. The estimated final volume was approximately 20–30 μl of sperm suspension (semen in addition to the culture medium), and the estimated sperm concentration was 101×10^6^±5×10^6^ spermatozoa ml^−1^ (mean±s.e.), ranging from 64×10^6^ to 145×10^6^ spermatozoa ml^−1^. The ambient temperature of the experimental room was 25°C, and the culture medium was prepared at this temperature one hour before the start of the experiment.

For thermal treatment experiments, we prepared three aliquots for each individual by combining 5 μl of sperm suspension with 45 μl of medium in separate microcentrifuge tubes (final volume: 50 μl per aliquot). Each aliquot was then incubated at one of the three experimental temperatures (28°C, 35°C, or 38°C; see section ‘Temperature treatments’) using precision thermal baths (Thermo Scientific Precision™). Sperm motility and velocity parameters were assessed immediately upon incubation initiation and subsequently at 30-min intervals over a 2-h incubation period.

### Temperature treatments

Following semen collection and preparation, samples from each male were exposed to three temperature treatments, representing ecologically relevant thermal conditions experienced by *P. extrilidus* in the wild (Gómez Alés et al., 2017, 2018). The first treatment, maintained at 28°C, approximated to operative environmental temperatures recorded in the field during the reproductive seasons in the Puna habitat of *P. extrilidus* (mean operative temperature 27.17°C; Gómez Alés et al., 2017). This temperature, therefore, represents the cooler environmental conditions commonly available to active individuals in their natural habitat. The second treatment, set at 35°C, corresponded to the preferred body temperature (T_pref_) of *P. extrilidus* during the reproductive and post-reproductive seasons (range 34.84–36.5°C; Gómez Alés et al., 2017), representing optimal thermal conditions for physiological function. The third treatment, maintained at 38°C, represents a supra-optimal temperature that induces thermal stress. Although this temperature causes decreased locomotor performance (Gómez Alés et al., 2018), it remains substantially below the critical thermal maximum for the species (CT_max_=44.45°C; Gómez Alés et al., 2018). These three temperatures collectively encompass the range of thermal conditions currently experienced by males and females in nature, including supra-optimal temperatures that may become increasingly frequent under projected climate-warming scenarios.

### Sperm parameters

Sperm motility and swimming kinematics were assessed by placing 10 μl of sperm suspension between slide and coverslip and recording at 10x magnification under a phase contrast microscope (Eclipse 50i Nikon) connected to a digital camera (Nikon Digital Sight DS-Fi2). For each sample and time point, we recorded an average of 5 to 10 microscopic fields per slide, with each field filmed for 5 s, in order to capture representative sperm populations. The samples were analyzed at room temperature (25°C) for a period of 50–60 s.

Video recordings were acquired at 4.6 frames s^−1^ and analyzed using the OpenCASA computer-assisted sperm analysis software (Alquézar-Baeta et al., 2019). The tracking parameters were configured as maximum particle size of 90 μm, minimum particle size of 30 μm, and connectivity set to 40. Individual sperm trajectories were tracked and the following kinematic parameters were calculated for each cell: VCL (μm s^−1^), VSL (μm s^−1^), VAP (μm s^−1^), linearity (LIN=VSL/VCL), straightness (STR=VSL/VAP), wobble (WOB=VAP/VCL), BCF (Hz), and fractal dimension (Fractal). Tracks with VAP values lower than 10 μm s^−1^ or LIN, STR, and WOB values of 100 were discarded from analysis, as these parameter values are characteristic of drifting immotile sperm or non-sperm particles. Additionally, the percentage of motile sperm was manually estimated for each sample (*n*=200 cells/sample) using ImageJ software (version 1.53t) with the Cell_Counter plugin (version 3.0.0).

### Data analysis

All analyses were conducted using R version 4.1.3 through RStudio (v2024.12.1) with α=0.05. The effects of temperature treatment, incubation time, and their interaction on sperm kinematic parameters were assessed via linear mixed-effects models. Models were fitted using the *lmer* and *glmer* functions of the *lme4* package (Bates et al., 2015) with binomial (MOT), gamma (VSL), normal (VCL, VAP, LIN, STR, and WOB), and lognormal (BCF) distributions. LIN, STR, and WOB values were transformed by calculating the arcsine of the square root of the proportion. Separate models were constructed for each sperm parameter using temperature (28°C, 35°C, 38°C), incubation time (0, 30, 60, 90, 120 min), and their interaction as categorical fixed effects. Individual male identity was included as a random intercept to account for between-individual variation in baseline sperm characteristics and to properly model the repeated-measures structure of the data (Zhang et al., 2025). The global significance (*P*-value) of each model fixed effects was tested by means of a likelihood ratio test (LRT) against a model excluding the factor of interest. Significant differences between levels of temperature and incubation time were analyzed via post-hoc estimated marginal means tests (*pairwise* function of the *emmeans* package).

## Supplementary Material


