# Supplementary material for: Post-ejaculatory thermal sensitivity of sperm performance in the high-altitude lizard Phymaturus extrilidus
Source: Biol Open. 2026 Jun 30;15(6):bio062678. doi: 10.1242/bio.062678 (PMC13382993; doi:10.1242/bio.062678)
Supplement: Supplementary file 1 [file biolopen-15-062678_review_history.pdf]

Post-ejaculatory thermal sensitivity of sperm performance in the high-altitude lizard Phymaturus extrilidus

Rodrigo Gómez Alés, Franco Valdez Ovallez, Maximiliano Tourmente and Guadalupe López Juri
10.1242/bio.062678

Editor: Nicholas Wu

Review timeline
Original submission: 10 May 2026
Editorial decision: 19 May 2026
First revision received: 27 May 2026
Accepted: 3 June 2026

Original submission

First decision letter

MS ID#: bio.062678

MS Title: Post-ejaculatory thermal sensitivity of sperm performance in the high-altitude lizard Phymaturus extrilidus

Authors: Rodrigo Gómez Alés; Guadalupe López Juri; Franco Valdez Ovallez; Maximiliano Tourmente

I have now reached a decision on the above manuscript.

The reviewer reports are shown at the bottom of this email.

As you will see, the reviewers gave favourable reports, but raised some critical points that will require amendments to your manuscript. I hope that you will be able to carry these out, because we would like to be able to accept your paper. I have also reviewed the paper and checked the reviewer comments if they were valid. Both reviewers and I found the paper to be of high quality with some minor clarifications about the methodology and results. Reviewer 2 made a good point about sperm extraction, preparation, and analysis at room temperature. Please clarify some of the points made about the potential confounding effects of the temperature exposure and duration during these phases for transparency. From my side, be more explicit about which variables or trait values were transformed and why. This gives a better understanding of the initial and final data structure.

At this stage, we also ask you to ensure your manuscript complies with our formatting guidelines - please see our manuscript preparation guidelines for details. Provided you are able to fully address the referees' comments, we are positive about publication of your paper (we accept over 95% of revision submissions) and therefore hope you won't mind any extra work involved in reformatting your manuscript at this point.

Please upload both a 'clean' version of your Word file, along with a highlighted version clearly showing where you have made changes in the revised manuscript. Please avoid using 'Track changes' in Word files as these are lost in PDF conversion.
I should be grateful if you would also provide a point-by-point response detailing how you have dealt with the points raised by the reviewers in the 'Response to Reviewers' box. Please attend to

© 2026. Published by The Company of Biologists under the terms of the Creative Commons Attribution License (https://creativecommons.org/licenses/by/4.0/).

all of the reviewers' comments. If you do not agree with any of their criticisms or suggestions please explain clearly why this is so.

Reviewer 1

Comments for the author

The study is very straightforward: assessing sperm function in a lizard in response to different incubation temperatures and durations. Given this simplicity, I do not have any major intellectual concerns to report. The manuscript is also very well written.

The experimental quality is good, with three testing temperatures that are biologically relevant. The methods are appropriate to investigate sperm function, and the statistical approaches are standard.

The number of replicates is good, especially as there are within-male replicates (i.e. repeated measures) for the incubation temperatures and durations. The methods are clear and comprehensive, with sufficient detail that permits reproducibility.

The conclusions are convincingly supported by the data. The experimental design is also convincing.

There is a good coverage of the literature in connection to the results and their interpretation.

Minor comments:

"in the Puna" in the Abstract will be confusing for many readers. At least, I would add "desert"

Lines 141-143, this statement is a bit confusing. If mating runs from March to April, how is mating observed in February supporting that knowledge?

Lines 145-146. This is the first time that the preferred temperature is mentioned. How was that temperature determined?

Line 148, "E = 0.65" will not mean much to many readers

Line 193: "male", and remove the comma after the parenthesis

Lines 246-239: this statement is about the "three temperatures". Will all those temperatures "become more frequent under projected climate change scenarios"? Or only the high extreme?

Line 274, remove the period after the parenthesis

Line 325, use the number of minutes instead of T4, as you don't use that notation anywhere else for what I could see

Lines 381-383. As summarized here, this other study (Quintero Pérez et al 2023) seems very similar to the one reported in the current manuscript. I think you could do a better job explaining how the two studies differ, and thus what's the novel contribution of the current study.

References: there are several issues here:

Remove the extra "de" in line 508
Some journal titles are capitalized, others are not; some are italicized, others are not
Some titles are capitalized, others are not
Lines 558-559, I would use only "Animals"
Line 703, scientific name in italics
Line 727, remove period after question sign

© 2026. Published by The Company of Biologists under the terms of the Creative Commons Attribution License (https://creativecommons.org/licenses/by/4.0/).

Figures: it would be useful to have a clear guide to the colors within the figures themselves, so that they can be read without having to go to the legend to know what the different lines represent.

Reviewer 2

Comments for the author

The manuscript entitled "Post-ejaculatory thermal sensitivity of sperm performance in the high-altitude lizard Phymaturus extrilidus" addresses an interesting and timely topic concerning the effects of temperature on sperm physiology in a high-altitude ectotherm. The study is relevant within the context of climate change biology and reproductive ecology, and the use of CASA-based sperm analysis represents a valuable methodological approach. The manuscript is generally well written and presents potentially important findings regarding the mismatch between somatic and reproductive thermal optima.

However, several pointsvshould be addressed before the manuscript can be considered for publication.

Line 126: please indicate that Phymaturus extrilidus exhibits internal fertilization and viviparity, rather than egg-laying as in many other reptiles. This information would be useful for readers unfamiliar with the biology of the study species.

Line 212: include the approximate ejaculate volume obtained by electroejaculation in this species. This information would help readers better understand sample handling and dilution procedures.

Line 213: indicate the sperm concentration and ejaculate volume of the collected samples, preferably as mean values and/or ranges.

Line 219: indicate the temperature of the sperm culture medium added during sample suspension preparation (10 and 20 µL). Please also specify the temperature of the medium used when preparing the 50 µL aliquots subsequently exposed to the different experimental temperatures. If the medium was maintained at room temperature (RT), please indicate the approximate RT and the duration for which the samples remained under these conditions before thermal exposure.

Line 239: indicate here the typical environmental temperature of the natural habitat of Phymaturus extrilidus, even if this information has been mentioned elsewhere in the manuscript. This would help readers contextualize the ecological relevance of the experimental temperatures used.

Line 252: specify the temperature conditions used during sperm observation between slide and coverslip. Indicate whether samples were analyzed at RT or maintained using a temperature-controlled microscope stage.

Line 254-255: if sperm analyses were performed at room temperature, please emphasize that the observation time was limited to 25-50 seconds, as prolonged exposure to room temperature could potentially affect sperm performance parameters. Alternatively, if a temperature-controlled microscope stage was used, please specify this information.
Line 267: similar to the previous point, specify whether analyses were performed at room temperature or using a temperature-controlled microscope stage.

Reviewer's Responses to Questions

Experimental quality
Are experiments performed using appropriate methods that will answer the question (or test the hypothesis or support the observations) posed by the authors? Is the right tool used for the job?
If 'No', please indicate reasons in Comments for Author box below.

© 2026. Published by The Company of Biologists under the terms of the Creative Commons Attribution License (https://creativecommons.org/licenses/by/4.0/).

Reviewer #1:
- Yes

Reviewer #2:
- Yes

Were the data analyzed using appropriate statistical tests?
If 'No', please indicate reasons in Comments for Author box below.
Reviewer #1:
- Yes

Reviewer #2:
- Yes

Reproducibility
Were experiments performed using adequate number of biological replicates?
If 'No', please indicate reasons in Comments for Author box below.
Reviewer #1:
- Yes

Reviewer #2:
- Yes

Is there sufficient raw data to assess the rigor of the analysis?
If 'No', please indicate reasons in Comments for Author box below.
Reviewer #1:
- Yes

Reviewer #2:
- Yes

Does the methods section provide sufficient detail to permit reproducibility?
If 'No', please indicate reasons in Comments for Author box below.
Reviewer #1:
- Yes

Reviewer #2:
- Yes

Completeness
Are the manuscript's conclusions supported by the data?
If 'No', please indicate reasons in Comments for Author box below.
Reviewer #1:
- Yes

Reviewer #2:
- Yes

Scholarship
Do the authors cite and discuss the merits of data that would argue for and against their conclusion?
If 'No', please indicate reasons in Comments for Author box below.
Reviewer #1:

© 2026. Published by The Company of Biologists under the terms of the Creative Commons Attribution License (https://creativecommons.org/licenses/by/4.0/).

- Yes

Reviewer #2:
- Yes

Does the manuscript title & abstract accurately reflect the contents of the manuscript, without hyperbole?
If 'No', please indicate reasons in Comments for Author box below.
Reviewer #1:
- Yes

Reviewer #2:
- Yes

First revision

Author response to reviewers' comments

Handling Editor

As you will see, the reviewers gave favourable reports, but raised some critical points that will require amendments to your manuscript. I hope that you will be able to carry these out, because we would like to be able to accept your paper. I have also reviewed the paper and checked the reviewer comments if they were valid. Both reviewers and I found the paper to be of high quality with some minor clarifications about the methodology and results. Reviewer 2 made a good point about sperm extraction, preparation, and analysis at room temperature. Please clarify some of the points made about the potential confounding effects of the temperature exposure and duration during these phases for transparency. From my side, be more explicit about which variables or trait values were transformed and why. This gives a better understanding of the initial and final data structure.

Dear Editor,

We are deeply grateful to you and the reviewers for the time and expertise invested in evaluating our manuscript. We sincerely appreciate your positive assessment and your interest in considering our manuscript for publication in Biology Open.

We have extensively revised the manuscript to incorporate the reviewers' recommendations, with particular attention to improving conceptual alignment, structural clarity, and precision in terminology. In addition, we have clarified which variables or trait values were transformed and the rationale behind these transformations. We hope these revisions improve the clarity of both the original and final data structure and facilitate a better understanding of our analyses and results.

Reviewer 1:

The study is very straightforward: assessing sperm function in a lizard in response to different incubation temperatures and durations. Given this simplicity, I do not have any major intellectual concerns to report. The manuscript is also very well written.

The experimental quality is good, with three testing temperatures that are biologically relevant. The methods are appropriate to investigate sperm function, and the statistical approaches are standard.

The number of replicates is good, especially as there are within-male replicates (i.e. repeated measures) for the incubation temperatures and durations. The methods are clear and

© 2026. Published by The Company of Biologists under the terms of the Creative Commons Attribution License (https://creativecommons.org/licenses/by/4.0/).

comprehensive, with sufficient detail that permits reproducibility.

The conclusions are convincingly supported by the data. The experimental design is also convincing.

There is a good coverage of the literature in connection to the results and their interpretation.

We appreciate your positive assessment of the experimental design and methodology. We are pleased that you recognized the rigor of our approach, particularly the inclusion of replicates for each male. We also appreciate your favorable evaluation of the results and conclusions presented in the manuscript.

Minor comments:

"in the Puna" in the Abstract will be confusing for many readers. At least, I would add "desert"

Line 55: We modified the sentence in the Abstract to clarify the geographic and ecological context by replacing "the Puna" with "the high-altitude Puna Desert".

Lines 141-143, this statement is a bit confusing. If mating runs from March to April, how is mating observed in February supporting that knowledge?

Lines 149-153: We thank the reviewer for pointing this out. We revised the sentence to clarify that Pizarro et al. (2022) inferred mating activity mainly between March and April based on gonadal evidence, whereas the observation reported by Noriega et al. (2023) suggests that mating may begin earlier, in February.

Lines 145-146. This is the first time that the preferred temperature is mentioned. How was that temperature determined?

Lines 154-155: We thank the reviewer for this observation. We clarified in the text that the preferred body temperature was experimentally determined using laboratory thermal gradients in Gómez Alés et al. (2017).

Line 148, "E = 0.65" will not mean much to many readers

Line 157: We removed the thermoregulatory index value (E = 0.65) from the sentence and retained the qualitative description of thermoregulatory behavior.

Line 193: "male", and remove the comma after the parenthesis

Line 198: We removed the comma.

Lines 246-239: this statement is about the "three temperatures". Will all those temperatures "become more frequent under projected climate change scenarios"? Or only the high extreme?

Lines 250-253: We thank the reviewer for this observation. We revised the sentence to clarify that only the supra-optimal temperature treatment (38°C), is the condition expected to become more frequent under projected climate-warming scenarios.

Line 274, remove the period after the parenthesis

Line 276: We removed the period.

Line 325, use the number of minutes instead of T4, as you don't use that notation anywhere else for what I could see

Line 324: We replaced "T4" with "120 min" for consistency with the notation used throughout the manuscript.

© 2026. Published by The Company of Biologists under the terms of the Creative Commons Attribution License (https://creativecommons.org/licenses/by/4.0/).

Lines 381-383. As summarized here, this other study (Quintero Pérez et al 2023) seems very similar to the one reported in the current manuscript. I think you could do a better job explaining how the two studies differ, and thus what's the novel contribution of the current study.

Lines 376-379: We thank the reviewer for this valuable observation. We revised this section to better distinguish our study from Quintero Pérez et al. (2023), emphasizing the novel contribution of our work, including the progressive time- dependent accumulation of post-ejaculatory thermal damage and its effects on sperm trajectory geometry in a high-altitude viviparous species.

References: there are several issues here: Remove the extra "de" in line 508
Some journal titles are capitalized, others are not; some are italicized, others are not Some titles are capitalized, others are not
Lines 558-559, I would use only "Animals" Line 703, scientific name in italics
Line 727, remove period after question sign

We have reviewed and corrected the references

Figures: it would be useful to have a clear guide to the colors within the figures themselves, so that they can be read without having to go to the legend to know what the different lines represent.

We understand your observation. We have added a color guide within the figures themselves to improve their interpretation.

<u>Reviewer 2: </u>The manuscript entitled "Post-ejaculatory thermal sensitivity of sperm performance in the high-altitude lizard Phymaturus extrilidus" addresses an interesting and timely topic concerning the effects of temperature on sperm physiology in a high- altitude ectotherm. The study is relevant within the context of climate change biology and reproductive ecology, and the use of CASA-based sperm analysis represents a valuable methodological approach. The manuscript is generally well written and presents potentially important findings regarding the mismatch between somatic and reproductive thermal optima.

However, several pointsvshould be addressed before the manuscript can be considered for publication.

Thank you very much for your comments and suggestions. We appreciate your positive evaluation of our work within the context of climate change biology and reproductive ecology, as well as your recognition of the relevance of the CASA-based sperm analysis. We have revised several sections of the manuscript in response to your recommendations and hope that these changes have substantially improved the work. We are very grateful for your contributions, as well as those of the other reviewer, which helped us strengthen and clarify the manuscript.

Line 126: please indicate that Phymaturus extrilidus exhibits internal fertilization and viviparity, rather than egg-laying as in many other reptiles. This information would be useful for readers unfamiliar with the biology of the study species.

Line 137: We clarified that *Phymaturus extrilidus* is a viviparous species with internal fertilization to provide additional biological context for readers unfamiliar with the reproductive biology of the genus.

Line 212: include the approximate ejaculate volume obtained by electroejaculation in this species. This information would help readers better understand sample handling and dilution procedures.

Lines 226-230: We appreciate the suggestion; however, we did not measure the exact ejaculate volume obtained from each male. During the electrostimulation procedure, we performed three sequential stimulation series and collected all semen expelled through the cloaca. Consequently, we cannot determine the precise total ejaculate volume for each individual. Our aim was for the final Eppendorf tube to contain 20 µl of culture medium together with all

© 2026. Published by The Company of Biologists under the terms of the Creative Commons Attribution License (https://creativecommons.org/licenses/by/4.0/).

collected semen, which generally resulted in a final volume consisting of approximately 20-30 µl of sperm suspension (semen in addition to the culture medium). We added this information to the manuscript.

Line 213: indicate the sperm concentration and ejaculate volume of the collected samples, preferably as mean values and/or ranges.

Lines 226-230: As mentioned previously, we do not know the sperm concentration or the exact ejaculate volume of the collected samples. Based on our experiments, we estimate that the final combined volume of culture medium and semen generally ranged between 20 and 30 µl; however, the precise ejaculate volume for each sample was not determined. We also estimated sperm concentration. Mean sperm concentration was 101 × 10^6 ± 5 × 10^6 spermatozoa mL^{-1} (mean ± SE), ranging from 64 × 10^6 to 145 × 10^6 spermatozoa mL^{-1}. We added this information to the manuscript.

Line 219: indicate the temperature of the sperm culture medium added during sample suspension preparation (10 and 20 µL). Please also specify the temperature of the medium used when preparing the 50 µL aliquots subsequently exposed to the different experimental temperatures. If the medium was maintained at room temperature (RT), please indicate the approximate RT and the duration for which the samples remained under these conditions before thermal exposure.

Line 229-230: We added the sentence "The ambient temperature of the experimental room was 25°C, and the culture medium was prepared at this temperature one hour before the start of the experiment" to clarify the conditions under which the culture medium was prepared. The ambient temperature of the experimental room was 25°C, which was also the temperature of both the culture medium and the medium used to prepare the 50 µL aliquots. These media were maintained under these conditions for approximately 1 hour prior to the start of the experiment.

Line 239: indicate here the typical environmental temperature of the natural habitat of *Phymaturus extrilidus*, even if this information has been mentioned elsewhere in the manuscript. This would help readers contextualize the ecological relevance of the experimental temperatures used.

Line 243-244: We clarified that the 28°C treatment corresponds to operative environmental temperatures commonly available in the Puna habitat during the reproductive season, emphasizing the ecological relevance of this experimental condition.

Line 252: specify the temperature conditions used during sperm observation between slide and coverslip. Indicate whether samples were analyzed at RT or maintained using a temperature-controlled microscope stage.

Line 259-260: We added the sentence "The samples were analyzed at room temperature (25°C) for a period of 50-60 seconds." to clarify the temperature conditions under which spermatozoa were observed between the slide and coverslip.

Line 254-255: if sperm analyses were performed at room temperature, please emphasize that the observation time was limited to 25-50 seconds, as prolonged exposure to room temperature could potentially affect sperm performance parameters. Alternatively, if a temperature-controlled microscope stage was used, please specify this information.

We addressed this point in the previous comment by adding to the manuscript sentence the time required for sperm observation and filming.

Line 267: similar to the previous point, specify whether analyses were performed at room temperature or using a temperature-controlled microscope stage.

In lines 259-260, we clarified the conditions under which the videos were recorded and subsequently used to measure all sperm parameters.

© 2026. Published by The Company of Biologists under the terms of the Creative Commons Attribution License (https://creativecommons.org/licenses/by/4.0/).

Second decision letter

MS ID#: bio.062678R1

MS Title: Post-ejaculatory thermal sensitivity of sperm performance in the high-altitude lizard Phymaturus extrilidus

Authors: Rodrigo Gómez Alés; Guadalupe López Juri; Franco Valdez Ovallez; Maximiliano Tourmente

I am happy to tell you that your manuscript has been accepted for publication in Biology Open, pending our standard publication integrity checks. It was accepted on 3rd June 2026.

© 2026. Published by The Company of Biologists under the terms of the Creative Commons Attribution License (https://creativecommons.org/licenses/by/4.0/).
